# COVID-19 and Cognitive Change in a Community-Based Cohort

**DOI:** 10.1001/jamanetworkopen.2025.18648

**Published:** 2025-06-30

**Authors:** Ryan T. Demmer, Talea Cornelius, Zarina Kraal, James R. Pike, Yifei Sun, Pallavi Balte, Chaoqi Wu, Norrina B. Allen, Mary Cushman, Astrid M. Suchy-Dicey, Mitchell S. V. Elkind, Virginia Howard, Anna Kucharska-Newton, Deb Levine, Pamela L. Lutsey, Jennifer Manly, Thomas H. Mosley, Priya Palta, Melinda C. Power, Sudha Seshadri, Russell P. Tracy, Keenan Walker, Josef Coresh, Elizabeth C. Oelsner

**Affiliations:** 1 Division of Epidemiology, Department of Quantitative Health Sciences, College of Medicine and Science, Mayo Clinic, Rochester, Minnesota; 2 Department of Epidemiology, Columbia University Mailman School of Public Health, New York, New York; 3 Division of Epidemiology and Community Health, School of Public Health, University of Minnesota, Minneapolis; 4 Department of Medicine, Columbia University Vagelos College of Physicians and Surgeons, New York, New York; 5 Department of Neurology, Columbia University Vagelos College of Physicians and Surgeons, New York, New York; 6 Department of Medicine, New York University Grossman School of Medicine, New York, New York; 7 Department of Biostatistics, Columbia University Mailman School of Public Health, New York, New York; 8 Department of Preventive Medicine, Northwestern University Feinberg School of Medicine, Chicago, Illinois; 9 Department of Medicine, Larner College of Medicine at the University of Vermont, Burlington; 10 Department of Pathology and Laboratory Medicine, Larner College of Medicine at the University of Vermont, Burlington; 11 Huntington Medical Research Institutes, Pasadena, California; 12 Department of Epidemiology, Columbia University Mailman School of Public Health, New York, New York; 13 Department of Epidemiology, School of Public Health, University of Alabama at Birmingham, Birmingham; 14 Department of Epidemiology, Gillings School of Global Public Health, University of North Carolina at Chapel Hill, Chapel Hill; 15 Department of Medicine, University of Michigan, Ann Arbor; 16 The Memory Impairment and Neurodegenerative Dementia (MIND) Center, University of Mississippi Medical Center, Jackson; 17 Department of Neurology, School of Medicine, University of North Carolina at Chapel Hill, Chapel Hill; 18 Department of Epidemiology, Milken Institute School of Public Health, George Washington University, Washington, DC; 19 Glenn Biggs Institute for Alzheimer’s and Neurodegenerative Diseases, Graduate School of Biomedical Sciences, University of Texas Health San Antonio, San Antonio; 20 Laboratory of Behavioral Neuroscience, National Institute on Aging, Baltimore, Maryland; 21 Division of Epidemiology, Department of Population Health, New York University Grossman School of Medicine, New York; 22 Optimal Aging Institute, New York University Grossman School of Medicine, New York

## Abstract

**Importance:**

SARS-CoV-2 infection has been linked to neurotoxic effects and cognitive deficits.

**Objective:**

To determine whether decreases in cognitive function were accelerated after SARS-CoV-2 infection compared with individuals not infected.

**Design, Setting, and Participants:**

Multicenter, prospective cohort study from 2016 to 2022 among 3525 participants alive on March 1, 2020, and enrolled in The Atherosclerosis Risk in Communities (ARIC) study and the Collaborative Cohort of Cohorts for COVID-19 Research study who completed a prepandemic cognitive assessment and a pandemic-era assessment of SARS-CoV-2 infection. Final analyses performed in November 2024.

**Exposure:**

SARS-CoV-2 infection determined via self-report of a positive SARS-CoV-2 test or health care professional diagnosis of COVID-19, a positive SARS-CoV-2 antinucleocapsid antibody response, or presence of an administrative code for COVID-19 on medical records.

**Main outcomes and measures:**

A neuropsychological battery assessed multiple cognitive domains, and a cocalibrated confirmatory factor analysis generated factor scores for global cognitive function. The primary outcome was the rate of excess change in cognitive function.

**Results:**

The 3525 eligible participants had a mean (SD) age of 80.8 (4.7) years, 2085 (59.1%) were female, 752 (21.4%) were Black, and 2773 (78.6%) were White. SARS-CoV-2 infection was detected among 307 participants (8.7%), 103 of whom (33.6%) were hospitalized. Among uninfected participants, the mean annualized change in cognitive function was −0.09 (95% CI, −0.13 to −0.04). Compared with this rate, change was faster (β = −0.06; 95% CI, −0.09 to −0.02) among participants hospitalized for infection, but not different from participants who were infected but not hospitalized (β = 0.00; 95% CI, −0.02 to 0.03). The association among participants hospitalized for infection was evident in the cognitive domains of memory and executive function, but not language.

**Conclusions and relevance:**

This cohort study of older participants found accelerated decreases in cognition among individuals hospitalized for SARS-CoV-2 infection, but not nonhospitalized infection, in comparison with individuals not yet infected.

## Key Points

**Question:**

Is SARS-CoV-2 infection and/or severity associated with acceleration in changes in cognitive function among older adults after accounting for important prepandemic confounders, including genetic risk for cognitive decline?

**Findings:**

In this cohort study of 3525 participants, cognitive function decreased more rapidly among participants hospitalized for a SARS-CoV-2 infection when compared with participants not infected with SARS-CoV-2. These findings were evident after robust multivariable adjustment for confounders.

**Meaning:**

These findings suggest that avoiding severe SARS-CoV-2 infection could help preserve cognitive function among older adults.

## Teaser

This cohort study evaluates whether the rate of change in cognitive function differed between individuals with vs without SARS-CoV-2 infection.

## Introduction

Persistent cognitive symptoms are frequently reported following SARS-CoV-2 infection.^1,2,3^ A recent report observed that 45% of patients with prior COVID-19 self-reported brain fog, poor memory, or reduced executive function continuing for at least 2 months after infection.^4^ To what extent SARS-CoV-2 infection causes accelerated loss of cognitive function, particularly among adults at elevated risk of dementia, is of clinical and public health concern.

While several studies have shown cognitive deficits following COVID-19,^5,6,7,8^ these reports have key limitations that are relevant to their internal and external validity. Many lacked objective preinfection cognitive assessments, which are necessary to untangle cause-effect relationships with respect to rate of change in cognition over time. Prior work in the UK BioBank illustrated how accounting for preinfection measures significantly altered the interpretation of the effect of infection on brain structure.^9^ Several studies lacked uninfected comparison groups, precluding control for the influence of pandemic period effects, such as social deprivation, which also could affect cognition. Many studies did not account for the confounding effects of prepandemic health behaviors, prevalent comorbidities, or apolipoprotein E (APOE) ε4 genotype, which has been previously reported to both increase risk for severe COVID-19^10^ and enhance infection-related risk for incident dementia.^11^ Finally, US-based studies have typically involved single center clinical samples (often case series) with limited racial diversity.^12,13,14,15,16^

We leveraged a multiracial US community-based sample of late-life adults with robust, longitudinal cognitive assessments, comprehensive confounder measurements, and systematic SARS-CoV-2 ascertainment to examine the association between infection history and short-term cognitive change. We hypothesized that SARS-CoV-2 infection would be associated with acceleration in cognitive change, accounting for prepandemic cognitive status and factors that may jointly be associated with infection susceptibility, cognition, and dementia risk.

## Methods

The Atherosclerosis Risk in Communities (ARIC)^17,18,19^ Study is a prospective cohort study that originally focused on the cause of atherosclerosis in a middle-aged sample of largely Black and White participants. Between 1987 and 1989, ARIC enrolled 15 792 participants from 4 US communities (Washington County, Maryland; Forsyth County, North Carolina; selected suburbs of Minneapolis, Minnesota; and Jackson, Mississippi). ARIC participants who were alive on March 1, 2020, and provided consent were eligible for SARS-CoV-2 ascertainment by the Collaborative Cohort of Cohorts for COVID-19 Research (C4R) Study. Participants or their legal representative provided written informed consent for all ARIC and C4R procedures. The ARIC and C4R studies were approved by the institutional review boards at all study sites. Follow-up rates in relation to cognitive assessments through ARIC visit 5 have been published.^20^ The present analysis uses visit 6 and visit 7 as the baseline; inclusion criteria for the present analysis included completion of a prepandemic cognitive assessment and assessment of SARS-CoV-2 infection status during the pandemic (eFigure 1 in Supplement 1). Strengthening the Reporting of Observational Studies in Epidemiology (STROBE) reporting guidelines were used in the development of this manuscript.

### SARS-CoV-2 Infection

Infection history was defined by a composite of (1) participant or proxy self-report of a positive SARS-CoV-2 test or health care professional diagnosis of COVID-19, ascertained via standardized questionnaires administered from May 13, 2020, to March 9, 2022; (2) positive SARS-CoV-2 antinucleocapsid antibody response, assessed via dried blood spot collected March 2 to August 6, 2021 (eMethods in Supplement 1); or (3) presence of the administrative code for COVID-19 (U07.1) in any position on medical records^19^ from the period of May 13, 2020, to March 9, 2022. Hospitalized infection was defined as an infection with (1) participant or proxy self-report of hospitalization for COVID-19, or (2) medical records for COVID-19 hospitalization (only 2 hospitalized infections were self-reported without a medical record confirmation) (eFigure 2 in Supplement 1). Sensitivity analyses were conducted after excluding cases identified via self-report alone (ie, no confirmation via a positive COVID-19 test, medical record adjudication, or serology).

### Cognitive Assessments

Details regarding the ARIC neuropsychological battery^20^ are provided in the eMethods in Supplement 1. Participants completed a prepandemic in-person cognitive assessment during ARIC visit 6 (2016-2017) or visit 7 (2018-2019) and a pandemic cognitive assessment during ARIC visit 8 (2020, modified phone-based assessment) and visit 9 (2021-2022, in-person) (eTable 1 in Supplement 1). All 3525 participants provided 1 prepandemic cognitive assessment and 2802 provided 1 pandemic era assessment (1009 at visit 8 and 1793 at visit 9). When multiple examinations were available, we preferentially used visit 7 (closest time point to the pandemic’s beginning) and visit 9 (assessments were in-person and it allowed for the longest possible follow-up time). Cocalibrated confirmatory factor analysis models^21,22^ generated factor scores for global cognitive function and for language, memory, and executive function domains.

### Covariates

As previously described,^23^ trained researchers collected covariates by administering validated questionnaires about participant self-reported: (1) demographics, including age, sex (male or female), race and center (race and center information were considered as proxies for socioeconomic status combined into 1 variable due to collinearity of race and center in ARIC; categories were Black, North Carolina; White, North Carolina; White, Maryland; White, Minnesota; Black, Mississippi), educational attainment (<high school, high school or equivalent, or >high school), health insurance status at baseline (present or absent as a proxy for socioeconomic status); (2) behavioral factors, including smoking and alcohol use history (both categorized as current, former, or never); (3) comorbidity history (present or absent at the last study visit) including diabetes, coronary heart disease, stroke, and hypertension. The TaqMan assay (Applied Biosystems) assessed APOE ε4 carrier status (defined as having ≥1 APOE ε4 risk alleles).^23^ All covariates were measured at either the study baseline visit in 1987 to 1989 (for non–time-varying characteristics) or the closest prepandemic examination (for time-varying characteristics: smoking, alcohol use, body mass index, blood pressure, and comorbidities).

### Statistical Analysis

We estimated the association between SARS-CoV-2 infection and cognitive score change using a linear mixed effects model that included infection status, time between cognitive assessments, and an interaction between infection and time. Fully adjusted models additionally included covariates and time × covariate interaction terms. A heterogeneous compound-symmetry variance-covariance matrix was used based on model fit. We explored effect modification by race-center, APOE genotype, diabetes, sex, education, and median age at baseline. We tested for differences between subgroups by performing an independent *t* test of model-based estimates of the rate of cognitive change in each stratum. Missing covariates (described in the prior section) were imputed using multiple imputation by chained equations. Ten imputed datasets were generated following 100 burn-in iterations. Parameter estimates from models fit to imputed data were combined using Rubin’s rule. eTable 2 in Supplement 1 presents characteristics of complete cases vs those with imputed data. Sensitivity analyses were conducted in complete cases only. All analyses were conducted using SAS version 9.4 (SAS Institute) in November 2024.

## Results

Table 1 presents prepandemic characteristics of the 3525 eligible participants, by infection status at the time of the pandemic cognition examination. Participants were a mean (SD) age of 80.8 (4.7) years, 2085 (59.1%) were female, 752 (21.4%) Black, and 2773 (78.6%) White. APOE ε4 allele carrier status did not differ by infection history. The proportion of individuals who developed infection was 8.7% (307 of 3525), of whom 33.6% (103 of 307) were hospitalized. The low number of infections was related to the relatively early timing of pandemic cognition assessment (90% before January 11, 2022). The median (range) time between baseline and follow-up cognitive assessments was 2.87 (0.65-6.10) years, and the median (range) time between date of infection and follow-up cognitive assessment was 0.78 (0.00-2.59) years. A total of 87.5% of participants were vaccinated, although only 55 infected individuals were vaccinated before their infection.

**Table 1.** Participant General Characteristics According to SARS-CoV-2 Status Among Participants Enrolled in the Atherosclerosis Risk in Communities Study and the Collaborative Cohort of Cohorts for COVID-19 Research

Variable	Participants, No. (%)			
	All (N = 3525)	No infection (n = 3218)	Infection without hospitalization (n = 204)	Infection with hospitalization (n = 103)
Age, y, mean (SD) (n = 3521)	80.8 (4.7)	80.8 (4.6)	80.3 (4.8)	81.2 (4.8)
Sex				
Male	1440 (40.9)	1317 (40.9)	72 (35.3)	51 (49.5)
Female	2085 (59.1)	1901 (59.1)	132 (64.7)	52 (50.5)
Race and center				
Black-Forsyth	73 (2.1)	65 (2.0)	3 (1.5)	5 (4.9)
Black-Jackson	679 (19.3)	617 (19.2)	34 (16.7)	28 (27.2)
White-Forsyth	794 (22.5)	731 (22.7)	45 (22.1)	18 (17.5)
White-Washington	923 (26.2)	811 (25.2)	82 (40.2)	30 (29.1)
White-Minneapolis	1056 (30.0)	994 (30.9)	40 (19.6)	22 (21.4)
Education (n = 3518)				
<High school	412 (11.7)	351 (10.9)	33 (16.2)	28 (27.2)
High school	1459 (41.5)	1329 (41.4)	87 (42.6)	43 (41.7)
>High school	1647 (46.8)	1531 (47.7)	84 (41.2)	32 (31.1)
APOE ε4 allele carrier (n = 3309)	908 (25.8)	835 (25.9)	49 (24.0)	24 (23.3)
Smoking status (n = 3076)				
Never smoker	1090 (35.4)	990 (35.3)	71 (38.8)	29 (33.7)
Former smoker	1807 (58.7)	1649 (58.7)	108 (59.0)	50 (58.1)
Current smoker	179 (5.8)	168 (6.0)	4 (2.2)	7 (8.1)
Alcohol use (n = 3496)				
Never drinker	771 (22.1)	673 (21.1)	69 (34.0)	29 (28.4)
Former drinker	1001 (28.6)	904 (28.3)	55 (27.1)	42 (41.2)
Current drinker	1724 (49.3)	1614 (50.6)	79 (38.9)	31 (30.4)
Body mass index (n = 3461), mean (SD)^a^	28.1 (5.6)	28.0 (5.6)	28.7 (5.5)	29.2 (5.9)
Systolic BP (n = 3504), mm Hg, mean (SD)	134.77 (19.4)	134.68 (19.5)	135.06 (18.9)	137.17 (18.7)
Hypertension (n = 3472)	2735 (77.6)	2492 (77.4)	160 (78.4)	83 (80.6)
Diabetes (n = 3402)	1137 (32.3)	1021 (31.7)	67 (32.8)	49 (47.6)
CHD (n = 3461)	556 (15.8)	514 (16.0)	23 (11.3)	19 (18.4)
Stroke (n = 3516)	175 (5.0)	158 (4.9)	12 (5.9)	5 (4.9)
Cognitive diagnosis (n = 3514)				
None	2664 (75.8)	2444 (76.2)	159 (78.7)	61 (59.2)
Mild cognitive impairment	576 (16.4)	533 (16.6)	23 (11.4)	20 (19.4)
Dementia	274 (7.8)	232 (7.2)	20 (9.9)	22 (21.4)
Length of time between assessments (n = 2763), mean (SD)	2.75 (0.93)	2.72 (0.93)	3.14 (0.72)	3.15 (0.90)
GCFS, mean (SD)^b^	−0.07 (0.95)	−0.05 (0.94)	−0.04 (0.94)	−0.48 (1.20)
Memory score (n = 3506), mean (SD)^b^	−0.01 (0.90)	−0.01 (0.90)	0.00 (0.91)	−0.23 (0.93)
Language score (n = 3520), mean (SD)^b^	−0.09 (0.87)	−0.08 (0.86)	−0.07 (0.88)	−0.43 (0.97)
Executive function score (n = 3434), mean (SD)^b^	−0.09 (0.88)	−0.09 (0.88)	−0.05 (0.88)	−0.34 (0.93)

Abbreviations: APOE, apolipoprotein E; BP, blood pressure; CHD, congestive heart failure; GCFS, global cognitive factor score.

^a^ Calculated as weight in kilograms divided by height in meters squared.

^b^ Cognitive scores assessed at baseline.

### SARS-CoV-2 and Global Cognitive Function

The annualized rate of change in global cognitive function (in SD units) was −0.09 (95% CI, −0.13 to −0.04) among uninfected participants and −0.10 (95% CI, −0.15 to −0.05) among infected participants (excess change among the infected, −0.01; 95% CI, −0.03 to 0.01) (eTable 3 in Supplement 1). Results were similar in a case-only analysis but when removing participants who only self-reported an infection without a second confirmatory source of information, the rate of excess change was modestly greater among infected individuals: −0.03 (95% CI, −0.05 to −0.00) (eTable 3 in Supplement 1). When separated by infection severity (Table 2), participants hospitalized for infection had a faster rate of annualized change (excess change, −0.06; 95% CI, −0.09 to −0.02), but participants with nonhospitalized infection did not (excess change, 0.00; 95% CI, −0.02 to 0.03). Results were materially unchanged in analyses of complete cases only or after excluding participants with an infection based on self-report alone (Table 2). Global cognitive function decreases were greater among participants for hospitalized infection than those with nonhospitalized infection (Table 2).

**Table 2.** Multivariable-Adjusted Association Between SARS-CoV-2 Severity and Change in Global Cognitive Function Score Among Participants Enrolled in the Atherosclerosis Risk in Communities Study and the Collaborative Cohort of Cohorts for COVID-19 Research

Result	Model 1 (unadjusted)		Model 2^a^	
	Annual change^b^	Excess annual change (vs no infection)^c^	Annual change^b^	Excess annual change (vs no infection)^c^
Imputation analysis (n = 3525)				
No infection	−0.09 (−0.10 to −0.09)	[Reference]	−0.09 (−0.13 to −0.04)	[Reference]
Infection, not hospitalized	−0.09 (−0.11 to −0.06)	0.01 (−0.02 to 0.03)	−0.08 (−0.13 to −0.03)	0.00 (−0.02 to 0.03)
Infection, hospitalized	−0.15 (−0.19 to −0.12)^d^	−0.06 (−0.10 to −0.02)	−0.14 (−0.20 to −0.09)^d^	−0.06 (−0.09 to −0.02)
Case only analysis (n = 2672)				
No infection	−0.09 (−0.10 to −0.09)	[Reference]	−0.10 (−0.14 to −0.05)	[Reference]
Infection, not hospitalized	−0.08 (−0.11 to −0.06)	0.01 (−0.01 to 0.04)	−0.09 (−0.14 to −0.04)	0.01 (−0.02 to 0.03)
Infection, hospitalized	−0.16 (−0.20 to −0.12)^d^	−0.06 (−0.11 to −0.02)	−0.15 (−0.21 to −0.09)^d^	−0.05 (−0.10 to −0.01)
Excluding 117 infections classified based on self-report alone^e^				
No infection	−0.09 (−0.10 to −0.09)	[Reference]	−0.09 (−0.13 to −0.04)	[Reference]
Infection, not hospitalized	−0.10 (−0.14 to −0.07)	−0.01 (−0.05 to 0.03)	−0.10 (−0.15 to −0.04)	−0.01 (−0.05 to 0.02)
Infection, hospitalized	−0.14 (−0.18 to −0.10)	−0.05 (−0.09 to −0.01)	−0.13 (−0.19 to −0.07)	−0.04 (−0.08 to −0.00)
Imputation analysis (n = 3525)^f^				
No infection	NA	NA	−0.08 (−0.12 to −0.04)	[Reference]
Infection, not hospitalized	NA	NA	−0.08 (−0.13 to −0.03)	0.00 (−0.02 to 0.03)
Infection, hospitalized	NA	NA	−0.13 (−0.19 to −0.07)^d^	−0.05 (−0.09 to −0.01)

Abbreviation: NA, not applicable.

^a^ Model 2 adjusts for age, sex, race-center, education, smoking, alcohol use, apolipoprotein E ε4 carrier status, body mass index, blood pressure, history of hypertension, coronary heart disease, diabetes, and stroke.

^b^ Annual change parameter estimates represent the overall annualized cognitive score change rate by infection status derived from the main effect of time + the interaction between time and infection status.

^c^ Excess annual change parameter estimates represent the excess change in cognitive scores associated with infection with or without hospitalization and are derived from the interaction of follow-up time with infection.

^d^ *P* < .05 for annual change in cognitive score between participants hospitalized for infection vs participants without hospitalized infection.

^e^ No additional confirmatory test.

^f^ Additional adjustment for baseline cognitive diagnosis.

### SARS-CoV-2 Infection and Cognitive Domains

As with global cognition, only hospitalized infection was associated with domain-specific cognitive score changes. The annualized rate of change in executive function and memory scores were −0.04 (95% CI, −0.08 to 0.01) and −0.02 (95% CI, −0.07 to 0.04) among uninfected participants and −0.07 (95% CI, −0.13 to −0.02) and −0.07 (95% CI, −0.14 to −0.00) among hospitalized infected participants, respectively (Table 3). The respective excess changes in executive function and memory scores among the hospitalized infected individuals were −0.04 (95% CI, −0.08 to −0.00) and −0.05 (95% CI, −0.10 to −0.00) (Table 3). Results were similar when comparing any infection vs no infection (eTable 4 in Supplement 1) in analyses limited to complete cases or after excluding participants with infections status based on self-report alone. Nonhospitalized infection was not associated with cognitive change in any domain.

**Table 3.** Association Between SARS-CoV-2 Infection and Change in Cognitive Domains of Memory, Language, and Executive Function Among 3525 Participants Enrolled in the Atherosclerosis Risk in Communities Study and the Collaborative Cohort of Cohorts for COVID-19 Research^a^

Cognitive domain	Imputation analysis among n = 3525		Case-only analysis among n = 2672^a^	
	Annual change^b^	Excess annual change (vs no infection)^c^	Annual change^b^	Excess annual change (vs no infection)^c^
Executive function				
No infection	−0.04 (−0.08 to 0.01)	[Reference]	−0.03 (−0.08 to 0.02)	[Reference]
Infection, not hospitalized	−0.04 (−0.09 to 0.01)	−0.01 (−0.03 to 0.02)	−0.04 (−0.10 to 0.01)	−0.01 (−0.04 to 0.02)
Infection, hospitalized	−0.07 (−0.13 to −0.02)	−0.04 (−0.08 to −0.00)	−0.08 (−0.15 to −0.02)	−0.05 (−0.09 to −0.01)
Memory				
No infection	−0.02 (−0.07 to 0.04)	[Reference]	−0.02 (−08 to 0.04)	[Reference]
Infection, not hospitalized	−0.01 (−0.07 to 0.05)	0.01 (−0.02 to 0.04)	0.00 (−0.07 to 0.07)	0.02 (−0.01 to 0.05)
Infection, hospitalized	−0.07 (−0.14 to 0.00)^d^	−0.05 (−0.10 to −0.00)	−0.07 (−0.15 to 0.01)	−0.05 (−0.10 to 0.01)
Language				
No infection	−0.04 (−0.09 to 0.01)	[Reference]	−0.03 (−0.09 to 0.02)	[Reference]
Infection, not hospitalized	−0.06 (−0.11 to −0.00)	−0.02 (−0.04 to 0.01)	−0.05 (−0.11 to 0.01)	−0.02 (−0.05 to 0.01)
Infection, hospitalized	−0.04 (−0.10 to 0.02)	0.00 (−0.04 to 0.04)	−0.02 (−0.10 to 0.05)	0.01 (−0.04 to 0.06)

^a^ Results adjusted for age, sex, race-center, education, smoking, alcohol use, apolipoprotein E ε4 carrier status, body mass index, blood pressure, history of hypertension, coronary heart disease, diabetes, and stroke.

^b^ Annual change parameter estimates represent the overall annualized cognitive score change rate by infection status derived from the main effect of time + the interaction between time and infection status.

^c^ Excess annual change parameter estimates represent the excess change in cognitive scores associated with infection with or without hospitalization and are derived from the interaction of follow-up time with infection.

^d^ *P* < .05 for annual change in cognitive score between participants hospitalized for infection vs participants without hospitalized infection.

### Effect Modification

Estimates stratified by race-center, APOE, diabetes, sex, education, and age are shown in the Figure and eTable 5 in Supplement 1. There was modest evidence for greater associations between hospitalized infection and cognitive change among participants with diabetes (vs no diabetes), individuals with less than a high school education (vs >high school), and among Black participants from the Forsyth County field center (vs White participants from the Forsyth County field center). Findings for race-center should be interpreted cautiously as there were only 5 hospitalized infections, producing wide confidence intervals, in the subgroup of Black participants from the Forsyth center (Table 1; eTable 5 in Supplement 1).

**Figure.**
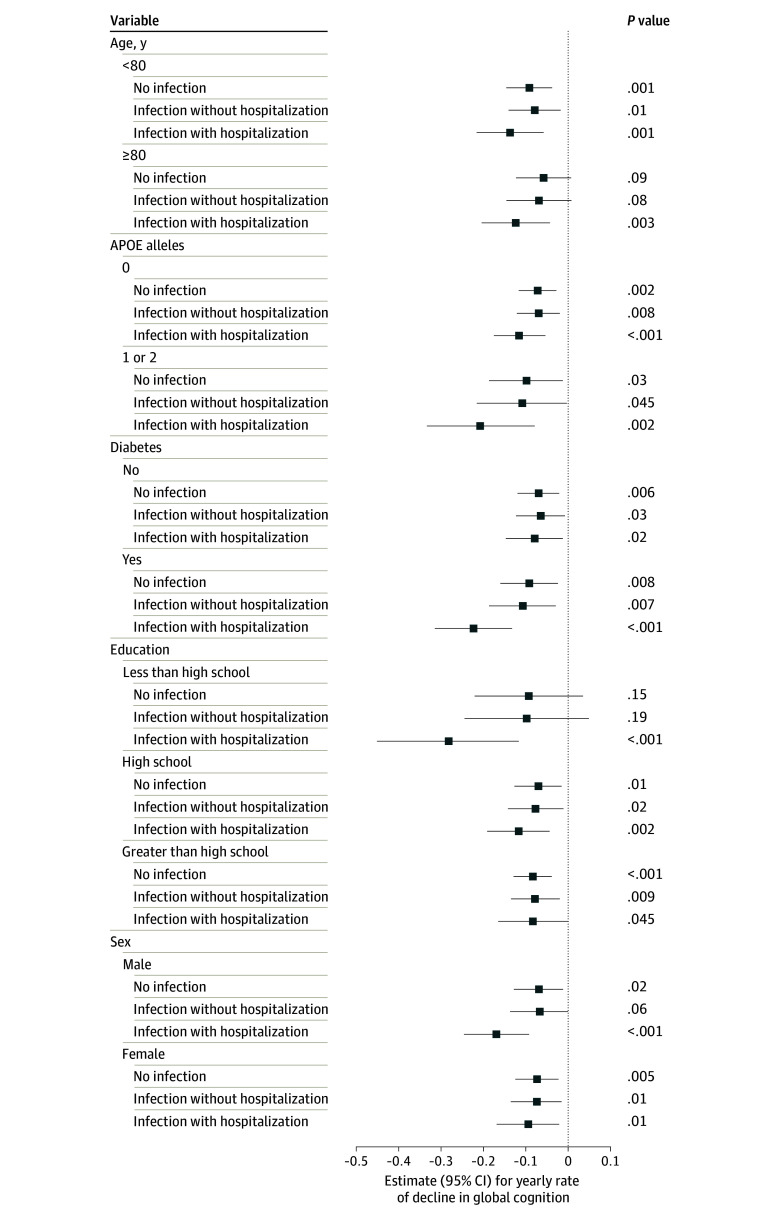
Association of SARS-CoV-2 Infection With Estimated Yearly Rate of Decline in Global Cognition Scores Stratified by Age, Education, Sex, Diabetes, and Apolipoprotein E (APOE) Genotype Results among 3525 participants enrolled in the Atherosclerosis Risk in Communities Study and the Collaborative Cohort of Cohorts for COVID-19 Research. Results supporting this figure are included in eTable 5 in Supplement 1.

## Discussion

In this multicenter, cohort study of Black and White older adults in the US, persons hospitalized for SARS-CoV-2 infection experienced larger decreases in global cognition than persons without prior infection. These findings were associated with excess decreases in memory and executive function. No meaningful acceleration in cognitive change was observed for the language domain. Cognitive change among persons with nonhospitalized SARS-CoV-2 infection was similar to that observed among persons without prior SARS-CoV-2 infection.

Prior studies conducted in Europe, South and Central America, Asia, and the US have reported cognitive deficits following SARS-CoV-2 infection.^5,24^ Our study supports a cognitive association with SARS-CoV-2 infection in cases requiring hospitalization, but not in milder cases managed at home (although analyses excluding infections based on self-report alone did suggest a modest association for overall infection). This nuance may be attributable to several important strengths of our study, which address existing knowledge gaps. First, most prior studies in the US were conducted within clinically based samples, with small sample sizes (<100 participants) and very limited confounder adjustment.^12,13,14,15,16^ Second, the largest study to date in the US that has reported associations between SARS-CoV-2 infection and cognitive deficits included only those with prior SARS-CoV-2 infection and compared cognition between participants with vs without evidence of postacute sequelae of COVID-19^25^ (PASC). Since PASC is often defined based on neurocognitive symptoms, treating PASC as the exposure of interest (vs infection without PASC) limits causal inferences due to bias resulting from shared criteria used to define both exposure and outcome. Third, most prior reports have often relied on a single cognitive assessment completed during the pandemic era without consideration of prepandemic cognitive status and therefore they could not directly study cognitive changes; our approach directly informs cognitive changes. Fourth, we accounted for cognition-relevant behavioral characteristics (eg, smoking and alcohol consumption), comorbidities (eg, cardiometabolic conditions), and APOE risk alleles. Our ability to account for the role of APOE ε4 genotype in this study is novel, as prior results on SARS-CoV-2 infection and cognition were potentially confounded by APOE genotype. The APOE ε4 genotype is associated with increased risk for viral infections,^26^ including SARS-CoV-2,^10^ and is also a causal risk factor for Alzheimer disease.^27^ Finally, unlike many prior studies, we included a large comparison group of individuals without known prior infection, supported by objective serological testing. This reduces the possibility that pandemic-related factors other than SARS-CoV-2 infection explained the more rapid longitudinal decreases in cognition observed among hospitalized individuals.

While our study did not confirm an association of nonhospitalized infection with short-term cognitive decline in older adults, potential causal effects of infection on cognition were not ruled out. Our negative findings are consistent with a prior study from the United Kingdom that observed cognitive deficits among nonhospitalized individuals with SARS-CoV-2 infection and persistent symptoms at 12 or more weeks (vs an infection-free comparator)^7^ but not among participants with infection symptoms resolving in less than 12 weeks. These findings suggest that mild infections do not contribute to long-term cognitive deficits. In contrast, a recent study from England did report cognitive deficits among individuals with symptoms persisting less than 12 weeks.^6^ However, the deficits observed for mild infections were modest and the sample size of more than 100 000 provided statistical power to detect small effects whereas our current study is not powered to detect equivalent effects. There are also many potential sources of heterogeneity in viral effects. These include different viral variants, vaccination status, antiviral and supportive treatments, and reinfections; our study was predominantly focused on first infections with pre-Omicron variants, often before the broad availability of antivirals. Although we did not adjust for vaccination status, more than 80% of infections occurred before vaccination in this cohort, reducing the potential for vaccination to meaningfully explain the associations observed for infection. Finally, cognitive effects of infection may be delayed beyond our study’s period of observation.

Although we showed a significant association between hospitalized infection and accelerated cognitive decline, it is important to acknowledge that prior work has shown that hospitalization for a range of infectious and noninfectious causes was associated with accelerated cognitive decline and incident dementia.^28,29,30^ Whether SARS-CoV-2 infection initiates pathobiological processes contributing to accelerated cognitive decline distinct from other causes of hospitalization remains unclear. It is possible that the physiological conditions enabling SARS-CoV-2 to progress to a severe state requiring hospitalization are the same conditions that increase susceptibility to accelerated cognitive change. Alternately, hospitalization-related factors such as pharmacological treatments, dietary changes, bed rest, or social isolation may contribute to cognitive changes. In this case, SARS-CoV-2 would act as an upstream causal factor, but its effects would be indirectly mediated through hospitalization-related mechanisms rather than directly mediated through viral exposure or immune response.

SARS-CoV-2 infection has a plausible biological link to reduced cognitive functioning. Prior studies suggest that SARS-CoV-2 can infect multiple tissues and cells across the body including brain tissue, and viral persistence among patients who have recovered from COVID-19 is associated with long COVID symptoms.^31,32,33,34,35,36^ The frequency of anosmia and ageusia, well-known hallmarks of SARS-CoV-2 infection, emphasizes the virus’ ability to impact neurological function. Moreover, numerous infections besides SARS-CoV-2 are linked to postacute infection syndromes,^37^ which include acute and chronic cognitive deficits.^38,39,40^ Substantial evidence links chronic infections, such as Epstein-Barr virus and HIV, to chronic autonomic dysfunction and neurodegenerative diseases.^41,42,43,44,45^ The potential role of β-amyloid in innate immunity provides another mechanistic explanation for our current findings given its potential antimicrobial effects against various pathogens. Some postulate that β-amyloid accumulation might be a protective acute response to pathogen colonization in the brain.^46,47,48,49^ However, prolonged infection could result in pathological β-amyloid accumulation resulting in diminished cognition. ARIC is currently conducting brain positron emission tomography and magnetic resonance imaging studies. Once completed, analyzing these data in reference to prior COVID-19 infection will help inform biological mechanisms.

We observed that the association between hospitalized SARS-CoV-2 and cognitive change was greater among people with diabetes, lower educational attainment, or Black participants from the Forsyth center. It is unclear what factors might explain these observations, and uncontrolled confounding cannot be excluded. For example, participants with lower education may have less social support and/or access to physical rehabilitation therapies following hospital discharge, limiting their recovery. Careful interpretation for education and race-center findings is necessary due to wide confidence intervals arising from the small number of hospitalized infections in key subgroups (eg, only 5 participants hospitalized for infection in the Black-Forsyth group). It is also possible that our assessments differentially estimate cognitive performance by race, although recent evidence suggests that the global cognitive function score is less susceptible to this bias.^50^ Nevertheless, future research and replication of these findings is necessary. In the case of diabetes, the potential for biological interaction exists, given the known impairments in cognition, immunity, and healing among people with diabetes. These heightened susceptibilities could synergize to produce accelerated cognitive changes. Whether these subgroup findings are true or artifactual requires further investigation.

### Limitations

This study has limitations. Misclassification of infections is possible despite our use of multiple sources of information about prior infection, including serology. For example, serological testing might have occurred before an individual’s infection or long after their infection when antibody levels waned and became undetectable. This would result in a misclassification of truly infected individuals as not infected. Missing data could also bias our findings, particularly among small subgroups (eg, participants with diabetes), although results from complete-case and multiple imputation analyses were consistent. Additionally, although our design is prospective, we do not know whether the modest relative reductions in cognitive scores following severe infection are transient or sustained because we do not have exact dates of infection for all participants. In ARIC, we observed that prepandemic hospitalized infections were associated with incident dementia during up to 32 years of follow-up, which supports the premise that cognitive changes following COVID-19 might persist.^11^ Longitudinal studies with longer follow-up are necessary to inform this question.

## Conclusions

In this study of a community-based, racially diverse cohort of older adults in the US, individuals hospitalized for SARS-CoV-2 infection, but not nonhospitalized SARS-CoV-2 infection, experienced accelerated decreases in global cognitive function scores. Our findings are consistent with prior work suggesting that severe SARS-CoV-2 infection might impact short-term cognition. Our null findings for nonhospitalized infection are consistent with some, but not all, of the prior literature, and warrant additional investigation in large, US general population-based cohorts with longitudinal data on cognition and potential confounders. Moreover, additional research is warranted to evaluate the effect of SARS-CoV-2 infection and reinfection on risk of long-term cognitive outcomes.

## Notes

Supplement 1.
eMethods.
**eTable 1.** Cognitive Tests Administered at Each ARIC Visit **eTable 2.** Baseline Characteristics Among Participants in the Case-Only Analysis vs Those With Missing Data for Whom Data Were Imputed **eTable 3.** Multivariable Adjusted Association Between SARS-CoV-2 Infection and Change in Global Cognitive Function Score Among Participants Enrolled in the Atherosclerosis Risk in Communities Study and the Collaborative Cohort of Cohorts for COVID-19 Research **eTable 4.** Association Between SARS-CoV-2 Infection and Change in Cognitive Scores for the Domains of Memory, Language, and Executive Function Among 3525 Participants Enrolled in the Atherosclerosis Risk in Communities Study and the Collaborative Cohort of Cohorts for COVID-19 Research **eTable 5.** Multivariable Adjusted Association Between SARS-CoV-2 Severity and Change in Global Cognitive Function Score Within Subgroups Defined by Age, Sex, Education, Race-Center, APOE ε4 Allele Carrier Status, and Diabetes Among Participants in the Atherosclerosis Risk in Communities Study and the Collaborative Cohort of Cohorts for COVID-19 Research **eFigure 1.** Flowchart of Participants Selected for, and Excluded From, the Analysis Among Those Who Attended Either Visit 6 or Visit 7 (2016-2019) and Contributed a Prepandemic Cognitive Assessment **eFigure 2.** Upset Plot Summarizing the Overlap of Sources of Information Used to Establish the Infection Exposure Definition
eReferences.

Supplement 2.
Data Sharing Statement

## References

[1] Jaywant A, Vanderlind WM, Alexopoulos GS, Fridman CB, Perlis RH, Gunning FM. Frequency and profile of objective cognitive deficits in hospitalized patients recovering from COVID-19. Neuropsychopharmacology. 2021;46(13):2235-2240. doi:10.1038/s41386-021-00978-8 33589778 PMC7884062

[2] Wanga V, Chevinsky JR, Dimitrov LV, . Long-term symptoms among adults tested for SARS-CoV-2 - United States, January 2020-April 2021. MMWR Morb Mortal Wkly Rep. 2021;70(36):1235-1241. doi:10.15585/mmwr.mm7036a1 34499626 PMC8437054

[3] Hirschtick JL, Titus AR, Slocum E, . Population-based estimates of post-acute sequelae of severe acute respiratory syndrome coronavirus 2 (SARS-CoV-2) infection (PASC) prevalence and characteristics. Clin Infect Dis. 2021;73(11):2055-2064. doi:10.1093/cid/ciab408 34007978 PMC8240848

[4] Perlis RH, Santillana M, Ognyanova K, . Prevalence and correlates of long COVID symptoms among US adults. JAMA Netw Open. 2022;5(10):e2238804. doi:10.1001/jamanetworkopen.2022.38804 36301542 PMC9614581

[5] Groff D, Sun A, Ssentongo AE, . Short-term and long-term rates of postacute sequelae of SARS-CoV-2 infection: a systematic review. JAMA Netw Open. 2021;4(10):e2128568. doi:10.1001/jamanetworkopen.2021.28568 34643720 PMC8515212

[6] Hampshire A, Azor A, Atchison C, . Cognition and memory after COVID-19 in a large community sample. N Engl J Med. 2024;390(9):806-818. doi:10.1056/NEJMoa2311330 38416429 PMC7615803

[7] Cheetham NJ, Penfold R, Giunchiglia V, . The effects of COVID-19 on cognitive performance in a community-based cohort: a COVID symptom study biobank prospective cohort study. EClinicalMedicine. 2023;62:102086. doi:10.1016/j.eclinm.2023.102086 37654669 PMC10466229

[8] Wood GK, Sargent BF, Ahmad ZU, . Post-hospitalisation COVID-19 cognitive deficits at one year are global and associated with elevated brain injury markers and grey matter volume reduction. Nat Med. 2024;31(1):245-257. doi:10.1038/s41591-024-03309-8 39312956 PMC11750706

[9] Douaud G, Lee S, Alfaro-Almagro F, . SARS-CoV-2 is associated with changes in brain structure in UK Biobank. Nature. 2022;604(7907):697-707. doi:10.1038/s41586-022-04569-5 35255491 PMC9046077

[10] Ostendorf BN, Patel MA, Bilanovic J, . Common human genetic variants of APOE impact murine COVID-19 mortality. Nature. 2022;611(7935):346-351. doi:10.1038/s41586-022-05344-2 36130725 PMC10957240

[11] Bohn B, Lutsey PL, Misialek JR, . Incidence of dementia following hospitalization with infection among adults in the atherosclerosis risk in communities (ARIC) study cohort. JAMA Netw Open. 2023;6(1):e2250126. doi:10.1001/jamanetworkopen.2022.50126 36622673 PMC9857407

[12] Hellmuth J, Barnett TA, Asken BM, . Persistent COVID-19-associated neurocognitive symptoms in non-hospitalized patients. J Neurovirol. 2021;27(1):191-195. doi:10.1007/s13365-021-00954-4 33528824 PMC7852463

[13] Whiteside DM, Oleynick V, Holker E, Waldron EJ, Porter J, Kasprzak M. Neurocognitive deficits in severe COVID-19 infection: case series and proposed model. Clin Neuropsychol. 2021;35(4):799-818. doi:10.1080/13854046.2021.1874056 33487098

[14] Hellgren L, Birberg Thornberg U, Samuelsson K, Levi R, Divanoglou A, Blystad I. Brain MRI and neuropsychological findings at long-term follow-up after COVID-19 hospitalisation: an observational cohort study. BMJ Open. 2021;11(10):e055164. doi:10.1136/bmjopen-2021-055164 34706965 PMC8551746

[15] Vannorsdall TD, Brigham E, Fawzy A, . Cognitive dysfunction, psychiatric distress, and functional decline after COVID-19. J Acad Consult Liaison Psychiatry. 2022;63(2):133-143. doi:10.1016/j.jaclp.2021.10.006 34793996 PMC8591857

[16] Becker JH, Lin JJ, Doernberg M, . Assessment of cognitive function in patients after COVID-19 infection. JAMA Netw Open. 2021;4(10):e2130645. doi:10.1001/jamanetworkopen.2021.30645 34677597 PMC8536953

[17] The ARIC investigators. The Atherosclerosis Risk in Communities (ARIC) study: design and objectives. The ARIC investigators. Am J Epidemiol. 1989;129(4):687-702. doi:10.1093/oxfordjournals.aje.a115184 2646917

[18] Thaweethai T, Jolley SE, Karlson EW, ; RECOVER Consortium. Development of a definition of postacute sequelae of SARS-CoV-2 infection. JAMA. 2023;329(22):1934-1946. doi:10.1001/jama.2023.8823 37278994 PMC10214179

[19] Wright JD, Folsom AR, Coresh J, . The ARIC (Atherosclerosis Risk In Communities) study: JACC focus seminar 3/8. J Am Coll Cardiol. 2021;77(23):2939-2959. doi:10.1016/j.jacc.2021.04.035 34112321 PMC8667593

[20] Knopman DS, Pike JR, Gottesman RF, . Patterns of cognitive domain abnormalities enhance discrimination of dementia risk prediction: the ARIC study. Alzheimers Dement. 2024;20(7):4559-4571. doi:10.1002/alz.13876 38877664 PMC11247695

[21] Gross AL, Power MC, Albert MS, . Application of latent variable methods to the study of cognitive decline when tests change over time. Epidemiology. 2015;26(6):878-887. doi:10.1097/EDE.0000000000000379 26414855 PMC4819068

[22] Crane PK, Narasimhalu K, Gibbons LE, . Item response theory facilitated cocalibrating cognitive tests and reduced bias in estimated rates of decline. J Clin Epidemiol. 2008;61(10):1018-27.e9. doi:10.1016/j.jclinepi.2007.11.011 18455909 PMC2762121

[23] Lu Y, Pike JR, Chen J, . Changes in Alzheimer disease blood biomarkers and associations with incident all-cause dementia. JAMA. 2024;332(15):1258-1269. doi:10.1001/jama.2024.6619 39068543 PMC11284635

[24] Crivelli L, Palmer K, Calandri I, . Changes in cognitive functioning after COVID-19: a systematic review and meta-analysis. Alzheimers Dement. 2022;18(5):1047-1066. doi:10.1002/alz.12644 35297561 PMC9073922

[25] Jaywant A, Gunning FM, Oberlin LE, . Cognitive symptoms of post-COVID-19 condition and daily functioning. JAMA Netw Open. 2024;7(2):e2356098. doi:10.1001/jamanetworkopen.2023.56098 38353947 PMC10867690

[26] Chen F, Ke Q, Wei W, Cui L, Wang Y. Apolipoprotein E and viral infection: risks and mechanisms. Mol Ther Nucleic Acids. 2023;33:529-542. doi:10.1016/j.omtn.2023.07.031 37588688 PMC10425688

[27] Fortea J, Pegueroles J, Alcolea D, . APOE4 homozygozity represents a distinct genetic form of Alzheimer’s disease. Nat Med. 2024;30(5):1284-1291. doi:10.1038/s41591-024-02931-w 38710950 PMC13310155

[28] Wilson RS, Hebert LE, Scherr PA, Dong X, Leurgens SE, Evans DA. Cognitive decline after hospitalization in a community population of older persons. Neurology. 2012;78(13):950-956. doi:10.1212/WNL.0b013e31824d5894 22442434 PMC3310309

[29] James BD, Wilson RS, Capuano AW, . Cognitive decline after elective and nonelective hospitalizations in older adults. Neurology. 2019;92(7):e690-e699. doi:10.1212/WNL.0000000000006918 30635482 PMC6382369

[30] Gracner T, Agarwal M, Murali KP, . Association of infection-related hospitalization with cognitive impairment among nursing home residents. JAMA Netw Open. 2021;4(4):e217528. doi:10.1001/jamanetworkopen.2021.7528 33890988 PMC8065379

[31] Crunfli F, Carregari VC, Veras FP, . Morphological, cellular, and molecular basis of brain infection in COVID-19 patients. Proc Natl Acad Sci U S A. 2022;119(35):e2200960119. doi:10.1073/pnas.2200960119 35951647 PMC9436354

[32] Gomes I, Karmirian K, Oliveira JT, . SARS-CoV-2 infection of the central nervous system in a 14-month-old child: a case report of a complete autopsy. Lancet Reg Health Am. 2021;2:100046. doi:10.1016/j.lana.2021.100046 34485969 PMC8397543

[33] Matschke J, Lütgehetmann M, Hagel C, . Neuropathology of patients with COVID-19 in Germany: a post-mortem case series. Lancet Neurol. 2020;19(11):919-929. doi:10.1016/S1474-4422(20)30308-2 33031735 PMC7535629

[34] Meinhardt J, Radke J, Dittmayer C, . Olfactory transmucosal SARS-CoV-2 invasion as a port of central nervous system entry in individuals with COVID-19. Nat Neurosci. 2021;24(2):168-175. doi:10.1038/s41593-020-00758-5 33257876

[35] Monje M, Iwasaki A. The neurobiology of long COVID. Neuron. 2022;110(21):3484-3496. doi:10.1016/j.neuron.2022.10.006 36288726 PMC9537254

[36] Zuo W, He D, Liang C, . The persistence of SARS-CoV-2 in tissues and its association with long COVID symptoms: a cross-sectional cohort study in China. Lancet Infect Dis. 2024;24(8):845-855. doi:10.1016/S1473-3099(24)00171-3 38663423

[37] Choutka J, Jansari V, Hornig M, Iwasaki A. Unexplained post-acute infection syndromes. Nat Med. 2022;28(5):911-923. doi:10.1038/s41591-022-01810-6 35585196

[38] Demmer RT, Norby FL, Lakshminarayan K, . Periodontal disease and incident dementia: the Atherosclerosis Risk in Communities Study (ARIC). Neurology. 2020;95(12):e1660-e1671. doi:10.1212/WNL.0000000000010312 32727837 PMC7713724

[39] Brown CH IV, Sharrett AR, Coresh J, . Association of hospitalization with long-term cognitive and brain MRI changes in the ARIC cohort. Neurology. 2015;84(14):1443-1453. doi:10.1212/WNL.0000000000001439 25762715 PMC4395884

[40] Warren-Gash C, Forbes HJ, Williamson E, . Human herpesvirus infections and dementia or mild cognitive impairment: a systematic review and meta-analysis. Sci Rep. 2019;9(1):4743. doi:10.1038/s41598-019-41218-w 30894595 PMC6426940

[41] Soldan SS, Lieberman PM. Epstein-Barr virus and multiple sclerosis. Nat Rev Microbiol. 2023;21(1):51-64. doi:10.1038/s41579-022-00770-5 35931816 PMC9362539

[42] Schreiner TG, Romanescu C, Schreiner OD, Nhambasora F. New insights on the link between Epstein-Barr virus infection and cognitive decline in neurodegenerative diseases (review). Exp Ther Med. 2024;28(5):413. doi:10.3892/etm.2024.12702 39268367 PMC11391170

[43] Carod-Artal FJ. Infectious diseases causing autonomic dysfunction. Clin Auton Res. 2018;28(1):67-81. doi:10.1007/s10286-017-0452-4 28730326

[44] Davis SE, Cirincione AB, Jimenez-Torres AC, Zhu J. The impact of neurotransmitters on the neurobiology of neurodegenerative diseases. Int J Mol Sci. 2023;24(20):15340. doi:10.3390/ijms242015340 37895020 PMC10607327

[45] Bjornevik K, Cortese M, Healy BC, . Longitudinal analysis reveals high prevalence of Epstein-Barr virus associated with multiple sclerosis. Science. 2022;375(6578):296-301. doi:10.1126/science.abj8222 35025605

[46] Lathe R, Schultek NM, Balin BJ, ; Intracell Research Group Consortium Collaborators. Establishment of a consensus protocol to explore the brain pathobiome in patients with mild cognitive impairment and Alzheimer’s disease: research outline and call for collaboration. Alzheimers Dement. 2023;19(11):5209-5231. doi:10.1002/alz.13076 37283269 PMC10918877

[47] Eimer WA, Vijaya Kumar DK, Navalpur Shanmugam NK, . Alzheimer’s disease-associated β-amyloid is rapidly seeded by herpesviridae to protect against brain infection. Neuron. 2018;99(1):56-63.e3. doi:10.1016/j.neuron.2018.06.030 30001512 PMC6075814

[48] Jorfi M, Maaser-Hecker A, Tanzi RE. The neuroimmune axis of Alzheimer’s disease. Genome Med. 2023;15(1):6. doi:10.1186/s13073-023-01155-w 36703235 PMC9878767

[49] Prosswimmer T, Heng A, Daggett V. Mechanistic insights into the role of amyloid-β in innate immunity. Sci Rep. 2024;14(1):5376. doi:10.1038/s41598-024-55423-9 38438446 PMC10912764

[50] Barnes LL, Yumoto F, Capuano A, Wilson RS, Bennett DA, Tractenberg RE. Examination of the factor structure of a global cognitive function battery across race and time. J Int Neuropsychol Soc. 2016;22(1):66-75. doi:10.1017/S1355617715001113 26563713 PMC4763720

