# Supplementary material for: COVID-19 and Cognitive Change in a Community-Based Cohort
Source: JAMA Netw Open. 2025 Jun 30;8(6):e2518648. doi: 10.1001/jamanetworkopen.2025.18648 (PMC12210084; doi:10.1001/jamanetworkopen.2025.18648)
Supplement: Supplement 1. — eMethods. eTable 1. Cognitive Tests Administered at Each ARIC Visit eTable 2. Baseline Characteristics Among Participants in the Case-Only Analysis vs Those With Missing Data for Whom Data Were Imputed eTable 3. Multivariable Adjusted Association Between SARS-CoV-2 Infection and Change in Global Cognitive Function Score Among Participants Enrolled in the Atherosclerosis Risk in Communities Study and the Collaborative Cohort of Cohorts for COVID-19 Research eTable 4. Association Between SARS-CoV-2 Infection and Change in Cognitive Scores for the Domains of Memory, Language, and Executive Function Among 3525 Participants Enrolled in the Atherosclerosis Risk in Communities Study and the Collaborative Cohort of Cohorts for COVID-19 Research eTable 5. Multivariable Adjusted Association Between SARS-CoV-2 Severity and Change in Global Cognitive Function Score Within Subgroups Defined by Age, Sex, Education, Race-Center, APOE ε4 Allele Carrier Status, and Diabetes Among Participants in the Atherosclerosis Risk in Communities Study and the Collaborative Cohort of Cohorts for COVID-19 Research eFigure 1. Flowchart of Participants Selected for, and Excluded From, the Analysis Among Those Who Attended Either Visit 6 or Visit 7 (2016-2019) and Contributed a Prepandemic Cognitive Assessment eFigure 2. Upset Plot Summarizing the Overlap of Sources of Information Used to Establish the Infection Exposure Definition eReferences. [file jamanetwopen-e2518648-s001.pdf]

Supplemental Online Content

Demmer RT, Cornelius T, Kraal Z. COVID-19 and accelerated cognitive change in a community-based cohort. *JAMA Netw Open*. 2025;8(7):e2518648. doi:10.1001/jamanetworkopen.2025.18648

eMethods.
eTable 1. Cognitive Tests Administered at Each ARIC Visit
eTable 2. Baseline Characteristics Among Participants in the Case-Only Analysis vs Those With Missing Data for Whom Data Were Imputed
eTable 3. Multivariable Adjusted Association Between SARS-CoV-2 Infection and Change in Global Cognitive Function Score Among Participants Enrolled in the Atherosclerosis Risk in Communities Study and the Collaborative Cohort of Cohorts for COVID-19 Research
eTable 4. Association Between SARS-CoV-2 Infection and Change in Cognitive Scores for the Domains of Memory, Language, and Executive Function Among 3525 Participants Enrolled in the Atherosclerosis Risk in Communities Study and the Collaborative Cohort of Cohorts for COVID-19 Research
eTable 5. Multivariable Adjusted Association Between SARS-CoV-2 Severity and Change in Global Cognitive Function Score Within Subgroups Defined by Age, Sex, Education, Race-Center, APOE ε4 Allele Carrier Status, and Diabetes Among Participants in the Atherosclerosis Risk in Communities Study and the Collaborative Cohort of Cohorts for COVID-19 Research
eFigure 1. Flowchart of Participants Selected for, and Excluded From, the Analysis Among Those Who Attended Either Visit 6 or Visit 7 (2016-2019) and Contributed a Prepandemic Cognitive Assessment
eFigure 2. Upset Plot Summarizing the Overlap of Sources of Information Used to Establish the Infection Exposure Definition
eReferences.

This supplemental material has been provided by the authors to give readers additional information about their work.

© 2025 Demmer RT et al. *JAMA Network Open*.

eMethods.

SAS Code for Statistical Models

Unadjusted model for the estimate of the influence of infection without hospitalization (‘Infection’) or infection with hospitalization (‘Hospitalization’) on rate of change in global cognitive score (‘Cognition’). The ‘Time_Between’ variable represents the time in years between the baseline and follow-up cognitive assessment. It can be interpreted as annualized rate of change in the global cognitive score. Interactions with this term can be interpreted as the additional annualized change in global cognitive score contributed by the interacting variable.

```
PROC MIXED DATA=data NOCLPRINT COVTEST NAMELEN=50;
CLASS id;
MODEL Cognition = Time_Between
Time_Between*Infection Time_Between*Hospitalization /SOLUTION COVB CL;
REPEATED / TYPE=csh SUBJECT=id;
BY _Imputation_;
ODS OUTPUT SOLUTIONF=Model1;
RUN;

PROC MIANALYZE PARMS(CLASSVAR=full)=Model1;
CLASS id;
MODELEFFECTS Time_Between
Time_Between*Infection Time_Between*Hospitalization;
RUN;
```

Multivariable adjusted model for the estimate of the influence of infection without hospitalization (‘Infection’) or infection with hospitalization (‘Hospitalization’) on rate of change in global cognitive score (‘Cognition’). The ‘Time_Between’ variable represents the time in years between the baseline and follow-up cognitive assessment. It can be interpreted as annualized rate of change in the global cognitive score. Interactions if this term with infection or hospitalized infection can be interpreted as the additional annualized change in global cognitive score contributed by those factors.

```
PROC MIXED DATA=data NOCLPRINT COVTEST NAMELEN=50;
CLASS id;
MODEL Cognition = Time_Between
Time_Between*Infection Time_Between*Hospitalization
Age Time_Between*Age Female Time_Between*Female Race1 Time_Between*Race1 Race 2 Time_Between*Race2 Race3
Time_Between*Race3 Race4 Time_Between*Race4
```

© 2025 Demmer RT et al. *JAMA Network Open*.

```
Education1 Time_Between*Education1 Education2 Time_Between*Education2
APOE Time_Between*APOE Smoke1 Time_Between*Smoke1 Smoke2 Time_Between*Smoke2
Alcohol1 Time_Between*Alcohol1 Alcohol2 Time_Between*Alcohol2 BMI Time_Between*BMI
SBP Time_Between*SBP Hypertension Time_Between*Hypertension CHD Time_Between*CHD
Diabetes Time_Between*Diabetes Stroke Time_Between*Stroke
/SOLUTION COVB CL;
REPEATED / TYPE=csh SUBJECT=id;
BY _Imputation_;
ODS OUTPUT SOLUTIONF=Model3;
RUN;

PROC MIANALYZE PARMS(CLASSVAR=full)=Model3;
CLASS id;
MODELEFFECTS Time_Between
Time_Between*Infection Time_Between*Hospitalization
Age Time_Between*Age Female Time_Between*Female Race1 Time_Between*Race1 Race 2 Time_Between*Race2 Race3
Time_Between*Race3 Race4 Time_Between*Race4
Education1 Time_Between*Education1 Education2 Time_Between*Education2
APOE Time_Between*APOE Smoke1 Time_Between*Smoke1 Smoke2 Time_Between*Smoke2
Alcohol1 Time_Between*Alcohol1 Alcohol2 Time_Between*Alcohol2 BMI Time_Between*BMI
SBP Time_Between*SBP Hypertension Time_Between*Hypertension CHD Time_Between*CHD
Diabetes Time_Between*Diabetes Stroke Time_Between*Stroke;
RUN;
```

Neuropsychological Battery

The neuropsychological battery administered in-person within the Atherosclerosis Risk in Communities (ARIC) study at Visits 5, 6, and 7 included the Mini Mental State Exam[1], Blessed scale[2,3], and ten cognitive tests.

1. *Digit Span Backwards (DSB)*[4]. A test of attention in which participants were read a string of numbers ranging from 2 to 7 digits. For each number string, the participant was asked to repeat the numbers backwards. Two trials were administered for each digit span length. The score ranged from 0 to 12 and documented the number of trials in which the participant correctly stated the reversed number string.

© 2025 Demmer RT et al. *JAMA Network Open*.

2. *Boston Naming Test (BNT)*[5]. A 30-item test that entailed naming common objects from a series of 30 line drawings. Participants were given 20 seconds to name the object in each drawing. The score ranged from 0 to 30 and indicated the number of objects correctly identified.

3. *Word Fluency Test (WFT)*[6]. A measure of phonemic fluency in which participants were given 60 seconds to state as many words as possible that began with the letters F, A, or S. 60 seconds were given for each letter. The score denoted the total number of acceptable words stated.

4. *Animal Naming Score (ANS)*[6]. A measure of semantic fluency that involved naming animals in 60 seconds. Names of extinct, imaginary, and magical animals were permitted. Credit was given for breeds, different names for males, females, or infants of the same species (e.g. bull, cow, calf) as well as superordinate and subordinate titles (e.g. dog and terrier). The score represents the total number of animals stated.

5. *Digit Symbol Substitution (DSS)*[4]. A test of executive functioning in which participants were asked to translate numbers to symbols using a key. The score is a count of numbers correctly translated to symbols within 90-seconds.

6. *Trail Making Test A (TMTA)*[7]. A test of processing speed in which participants were presented with numbers ranging from 1 to 25. Each number was placed in a separate circle and distributed haphazardly across a page. Participants were asked to draw lines connecting the numbers sequentially. The score indicated the number of seconds the participant took to complete the test. Participants who took longer than four minutes or who made more than 5 errors were given the maximum score of 240 seconds.

7. *Trail Making Test B (TMTB)*[7]. A variation of the TMTA in which participants were presented with numbers ranging from 1 to 13 and letters ranging from A to L. Participants were asked to draw lines connecting the numbers and letters in a sequential, alternating fashion. The score represents the number of seconds required for the participant to complete the test and was calculated using the same rules applied to the TMTA.

8. *Incidental Learning (ILR)*[8]. A test of delayed recall. Immediately following completion of the DSS, participants were asked to write down as many symbols as they could remember followed by the number paired with each symbol. The score ranged from 0 to 9 and separately documented the number of symbols and symbol-pairs recalled.

9. *Logical Memory Test (LMT)*[4]. A test of recall from two short stories read aloud to the participant. Initially, participants were asked to recall the details immediately following the reading of each story. Participants were then informed there would be additional questions about these stories at a subsequent point in time. After a delay of approximately 20 minutes, the participants were again asked to recall the details of each story. The score reflected the number of details correctly recalled with a maximum score of 25 per story.

© 2025 Demmer RT et al. *JAMA Network Open*.

10. *Delayed Word Recall (DWR)*[9]. A 10-word test of delayed episodic verbal memory in which participants were presented with 10 common nouns that they were asked to use in a sentence. After a five-minute delay, participants were given 60 seconds to recall the words. The score ranged from 0 to 10 and cataloged the number of words correctly recalled.

The in-person assessment protocol was initiated in January 2020 for Visit 8 but stopped in March 2020 due to the coronavirus pandemic. A modified phone-based protocol was implemented between July and December 2020. The phone-based battery comprised six tests.

1. *Digit Span Backwards (DSB)*[8]. A test of attention identical to the version administered in-person.

2. *Word Fluency Test (WFT)*[6]. A measure of phonemic fluency identical to the in-person version but limited to the letters F and A.

3. *Animal Naming Score (ANS)*[6]. A measure of semantic fluency identical to the version administered in-person.

4. *Oral Trail Making Test A (TMTA)*[10]. An oral version of the face-to-face test [7] in which participants were asked to recite the numbers 1 through 25. The score documented the number of seconds the participant took to complete this task. Participants who took longer than four minutes or who made more than 5 errors were given the maximum score of 240 seconds.

5. *Oral Trail Making Test B (TMTB)*[10]. An oral task in which participants were asked to verbally alternate between the numbers 1 through 13 and the letters A through L. The score denoted the number of seconds required to complete the task and was calculated using the same rules applied to the TMTA.

6. *Consortium to Establish a Registry for Alzheimer's Disease Word List (CERAD)*[11]. A 10-word test of immediate and delayed episodic verbal memory. The test comprised four trials. During the first three trials, participants were read ten common nouns and asked to repeat as many words as they could remember. The order in which the words were stated to the participant varied with each trial. The fourth trial was administered several minutes later. The score for each trial ranged from 0 to 10 and represented the number of words correctly remembered.

Available cognitive tests at each visit were used to compute a factor score of global cognitive function for each participant[12] that was standardized to ARIC Visit 5. A global factor score was chosen over other summary measures, such as weighted averages, since it mitigates measurement error, improves precision, has interval-level properties, and has minimal floor or ceiling effects. Comparable factor scores were calculated for the cognitive domains[13] of language (BNT, WFT, and ANS), executive function (DSS, TMTA, and TMTB), and memory (ILR, LMT, DWR, and CERAD).

© 2025 Demmer RT et al. *JAMA Network Open*.

A detailed description of the factor scores, including the code needed for replication, has been previously published[14]. Factor scores for the domains of memory, language, and executive function were computed as previously described[14]. However, a change was made to the global cognition factor score. To reduce missingness, co-calibration was used to compute a global cognition factor score from both in-person tests and phone-based tests administered during the pandemic. Co-calibration can be performed to link test batteries across assessment modalities when one or more cognitive tests are psychometrically equivalent across modalities[15]. These tests are used as linking items to produce scores for cognitive function that are comparable even if the battery of cognitive tests varies over time[14,16]. For ARIC, the tests selected as linking items were the digit span backwards, word fluency test, and animal naming score.

Serological Assays

Serological assays were performed, as previously described[17,18]. Briefly, the dried blood spot (DBS) requires that several drops of whole blood from a finger prick or blood collection tube be absorbed into a bar-coded, Whatmann filter paper card. Participants who consented to the serosurvey completed the DBS at an in-person exam or at home and returned the sample to a central laboratory via United States Postal mail. Serological assays were performed on DBS eluates by the Wadsworth Center, New York State Department of Health (Albany, NY, USA), using validated methods[18]. The assays were designed to detect IgG for SARS-CoV-2 S1 protein, which may be induced by natural infection or currently approved COVID-19 vaccines, and nucleocapsid (N) protein, which is induced by natural infection only. IgG reactivity to N protein or S1 protein among the unvaccinated were considered to have a history of infection.

Definition of Mild Cognitive Impairment and Dementia

When a cognitive examination detected cognitive impairment, an informant interview was conducted. The informant interview included the Clinical Dementia Rating scale[19] and Functional Activities Questionnaire[20]. When a reliable informant could not be identified, the Clinical Dementia Rating scale and Functional Activities Questionnaire were administered to the participant. A computer algorithm generated a preliminary diagnosis of no impairment, mild cognitive impairment, or dementia based on the cognitive examination, Clinical Dementia Rating scale, and Functional Activities Questionnaire. The algorithmic diagnosis was reviewed by an expert panel of clinicians and neuropsychologists. One clinician and one neuropsychologist independently rendered a diagnosis for each participant. Discordant cases were assigned to an adjudicator for final determination.

Among participants who did not complete a cognitive examination, incident dementia was ascertained from a Telephone Interview for Cognitive Status-Modified[20-22] administered to the participant and adjusted for education, an informant interview comprising the Clinical Dementia Rating scale and Functional Activities Questionnaire, an Ascertain Dementia Eight-Item Informant Questionnaire[23], or a phone-based Six-Item Cognitive Screener[24] administered to the participant. If the participant was lost to follow-up or deceased, hospitalization discharge codes and diagnostic codes from death certificates were used to identify incident dementia[25,26]. When dementia was identified through an informant interview, hospitalization record, or death certificate, the date of onset was estimated

© 2025 Demmer RT et al. *JAMA Network Open*.

as 180 days before the diagnosis or interview. Participants without a dementia diagnosis were censored at the latest available assessment, interview, or hospitalization record. Deceased participants without dementia were censored 180 days prior to death.

© 2025 Demmer RT et al. *JAMA Network Open*.

eTable 1. Cognitive Tests Administered at Each ARIC Visit

	Visit 6 (2016-17)	Visit 7 (2018-19)	Visit 8* (2020)	Visit 9 (2021-22)
Digit Span Backwards	✓	✓	✓	✓
Boston Naming Test	✓	✓		✓
Word Fluency Test, Letter F	✓	✓	✓	✓
Word Fluency Test, Letter A	✓	✓	✓	✓
Word Fluency Test, Letter S	✓	✓		✓
Animal Naming Score	✓	✓	✓	✓
Digit Symbol Substitution	✓	✓		✓
Trail Making Test A	✓	✓	✓	✓
Trail Making Test B	✓	✓	✓	✓
Delayed Word Recall	✓	✓		✓
Incidental Learning, Symbols Recalled	✓	✓		✓
Incidental Learning, Digit-Symbol Pairs Recalled	✓	✓		✓
Logical Memory Test, Story A, Immediate Recall	✓	✓		✓
Logical Memory Test, Story B, Immediate Recall	✓	✓		✓
Logical Memory Test, Story A, Delayed Recall	✓	✓		✓
Logical Memory Test, Story B, Delayed Recall	✓	✓		✓
CERAD Word List, Immediate Recall Trial 1			✓	
CERAD Word List, Immediate Recall Trial 2			✓	
CERAD Word List, Immediate Recall Trial 3			✓	
CERAD Word List, Delayed Recall			✓	

Abbreviations: ARIC-NCS, Atherosclerosis Risk in Communities Cognitive Study; CERAD, Consortium to Establish a Registry for Alzheimer's Disease; COVID, Coronavirus Disease. *Visits 8 was phone-based to accommodate pandemic-related restrictions on in-person examinations.

© 2025 Demmer RT et al. *JAMA Network Open*.

eTable 2. Baseline Characteristics Among Participants in the Case-Only Analysis vs Those With Missing Data for Whom Data Were Imputed

	**Complete Case (n = 2,672)**	**Imputed Case (n = 853)**	**p-value**
	Mean (SD) or N (%)	Mean (SD) or N (%)	
Age (years)	80.65 (4.51)	81.23 (5.07)	0.002
**Sex**			<0.001
Male (%)	1,136 (42.5%)	304 (35.6%)	
Female (%)	1,536 (57.5%)	549 (64.4%)	
**Race/Ethnicity**			<0.001
Black-Forsyth (%)	41 (1.5%)	32 (3.8%)	
White-Forsyth (%)	568 (21.3%)	226 (26.5%)	
Black-Jackson (%)	469 (17.6%)	210 (24.6%)	
White-Washington Co. (%)	728 (27.2%)	195 (22.9%)	
White-Minneapolis (%)	866 (32.4%)	190 (22.3%)	
**Education**			0.16
<High School (%)	298 (11.2%)	114 (13.5%)	
High School (%)	1,109 (41.5%)	350 (41.4%)	
>High School (%)	1,265 (47.3%)	382 (45.2%)	
1 or 2 APOE ε4 Alleles (%)	748 (28.0%)	160 (25.1%)	0.14
**Smoking Status**			0.70
Never Smoker (%)	953 (35.7%)	137 (33.9%)	
Former Smoker (%)	1,562 (58.5%)	245 (60.6%)	
Current Smoker (%)	157 (5.9%)	22 (5.4%)	
**Alcohol Use**			<0.001
Never Drinker (%)	530 (19.8%)	241 (29.2%)	
Former Drinker (%)	759 (28.4%)	242 (29.4%)	
Current Drinker (%)	1,383 (51.8%)	341 (41.4%)	
Body Mass Index (kg/m^2)	28.07 (5.36)	28.15 (6.18)	0.75
Systolic BP (mm Hg)	134.36 (19.23)	136.11 (19.96)	0.023
Hypertension (%)	2,020 (78.2%)	645 (80.6%)	0.14
Diabetes (%)	858 (32.1%)	279 (38.2%)	0.002
CHD (%)	434 (16.2%)	122 (15.5%)	0.60
Stroke (%)	131 (4.9%)	44 (5.2%)	0.72
**Cognitive Diagnosis**			<0.001
None (%)	2,083 (78.0%)	581 (69.0%)	
Mild Cognitive Impairment (%)	415 (15.5%)	161 (19.1%)	
Dementia (%)	174 (6.5%)	100 (11.9)	
GCFS	0.02 (0.90)	-0.32 (1.06)	<0.001
Memory score	0.04 (0.88)	-0.19 (0.94)	<0.001
Language score	-0.03 (0.85)	-0.27 (0.90)	<0.001
Executive Function score	-0.03 (0.87)	-0.31 (0.90)	

Data were imputed for the following variables with missing data: age (n=4), Education (n=7), smoking status (n=449), alcohol (n=29), APOE (n=216), cognitive diagnosis (n=11), BMI (n=65), SBP (n=21), Hypertension (n=53), Diabetes (n=123), CHD (n=64), Stroke (n=9), Memory score (n=19), Language score (n=5), Executive function score (n=91)

© 2025 Demmer RT et al. *JAMA Network Open*.

eTable 3. Multivariable Adjusted Association Between SARS-CoV-2 Infection and Change in Global Cognitive Function Score Among Participants Enrolled in the Atherosclerosis Risk in Communities Study and the Collaborative Cohort of Cohorts for COVID-19 Research

	Annual Change	Excess Annual Change (vs. No Infection)
Results from imputation analysis among n=3,525 participants		
**No Infection**	-0.09 (-0.13, -0.04)	
**Infection**	-0.10 (-0.15, -0.05)	-0.01 (-0.03, 0.01)
Results from complete case only analysis among n=2,672 participants		
**No Infection**	-0.10 (-0.14, -0.05)	
**Infection**	-0.11 (-0.16, -0.06)	-0.01 (-0.03, 0.02)
Results including only definite infections (excluding n=117 infections without expert adjudication or a positive COVID test)		
**No Infection**	-0.09 (-0.13, -0.04)	
**Infection**	-0.11 (-0.16, -0.06)	-0.03 (-0.05, -0.00)*

Adjusts for age, sex, race-center, education, smoking, alcohol use, APOE ε4 carrier status, BMI, blood pressure, history of hypertension, coronary heart disease, diabetes, stroke. ‘Annual Change’ parameter estimates represent the overall annualized rate of change in cognitive scores by infection status derived from the main effect of time + the interaction between time and infection status. ‘Excess Annual Change’ parameter estimates represent the excess change in cognitive scores associated with infection (with or without hospitalization) and these estimates are derived from the term for the interaction of follow-up time with infection status.

*p=0.058 for excess annual change in executive function score associated with infection in the sensitivity analysis including only definite infections.

© 2025 Demmer RT et al. *JAMA Network Open*.

eTable 4. Association Between SARS-CoV-2 Infection and Change in Cognitive Scores for the Domains of Memory, Language, and Executive Function Among 3525 Participants Enrolled in the Atherosclerosis Risk in Communities Study and the Collaborative Cohort of Cohorts for COVID-19 Research

	Annual Change	Excess Annual Change (vs. No Infection)
	**Memory**	
**No Infection**	-0.02 (-0.08, 0.04)	
**Infection**	-0.03 (-0.09, 0.03)	-0.01 (-0.03, 0.02)
	**Language**	
**No Infection**	-0.04 (-0.09, 0.01)	
**Infection**	-0.05 (-0.11, -0.00)	-0.01 (-0.03, 0.01)
	**Executive Function**	
**No Infection**	-0.04 (-0.08, 0.01)	
**Infection**	-0.06 (-0.10, -0.00)	-0.02 (-0.04, 0.00)

Results adjusted for age, sex, race-center, education, smoking, alcohol use, APOE ε4 carrier status, BMI, blood pressure, history of hypertension, coronary heart disease, diabetes, stroke. 'Annual Change' parameter estimates represent the overall annualized rate of change in cognitive scores by infection status derived from the main effect of time + the interaction between time and infection status. 'Excess Annual Change' parameter estimates represent the excess change in cognitive scores associated with infection (with or without hospitalization) and these estimates are derived from the term for the interaction of follow-up time with infection status.

© 2025 Demmer RT et al. *JAMA Network Open*.

eTable 5. Multivariable Adjusted Association Between SARS-CoV-2 Severity and Change in Global Cognitive Function Score Within Subgroups Defined by Age, Sex, Education, Race-Center, APOE ε4 Allele Carrier Status, and Diabetes Among Participants in the Atherosclerosis Risk in Communities Study and the Collaborative Cohort of Cohorts for COVID-19 Research				
**Age**				
	<80 Years		*≥80 Years*	
	Annual Change	**Excess Annual Change**	**Annual Change**	**Excess Annual Change**
**No Infection**	-0.09 (-0.15, -0.04) p=0.001, n=1,491	*Reference*	-0.06 (-0.12, 0.01) p=0.089, n=1,727	*Reference*
**Infection, not hospitalized**	-0.08 (-0.14, -0.02) p=0.012, n=104	0.01 (-0.02, 0.04) p=0.42	-0.07 (-0.14, 0.01) p=0.076, n=100	-0.01 (-0.05, 0.03) p=0.55
**Infection, hospitalized**	-0.14 (-0.22, -0.06) p=0.001, n=40	-0.05 (-0.10, 0.01) p=0.12	-0.12 (-0.20, -0.04) p=0.003, n=63	-0.07 (-0.12, -0.01) p=0.012
**Sex**				
	Females		*Males*	
**No Infection**	-0.07 (-0.12, -0.02) p=0.005, n=1,901	*Reference*	-0.07 (-0.13, -0.01) p=0.022, n=1,317	*Reference*
**Infection, not hospitalized**	-0.08 (-0.13, -0.02) p=0.013, n=132	-0.00 (-0.03, 0.03) p=0.89	-0.07 (-0.14, 0.00) p=0.062, n=72	0.00 (-0.04, 0.04) p=0.91
**Infection, hospitalized**	-0.09 (-0.17, -0.02) p=0.012, n=52	-0.02 (-0.08, 0.03) p<0.001	-0.17 (-0.25, -0.09)* p<0.0001, n=51	-0.10 (-0.15, -0.05)† p<0.001
**Education**				
	< High School		*High School*	
**No Infection**	-0.09 (-0.22, 0.04) p=0.15, n=351	*Reference*	-0.07 (-0.13, -0.01) p=0.13, n=1,329	*Reference*
**Infection, not Hospitalized**	-0.10 (-0.07, 0.08) p=0.19, n=33	-0.01 (-0.08, 0.07) p=0.89	-0.08 (-0.14, -0.01) p=0.023, n=87	-0.01 (-0.04, 0.03) p=0.73
**Infection, Hospitalized**	-0.28 (-0.45, -0.12)* p<0.001, n=28	-0.19 (-0.30, -0.09) p<0.001	-0.12 (-0.19, -0.04) p=0.002, n=43	-0.05 (-0.10, -0.00)† p=0.07
	> High School			
**No Infection**	-0.08 (-0.13, -0.04) p<0.001, n=1,531	*Reference*		
**Infection, not Hospitalized**	-0.08 (-0.13, -0.02) p=0.009, n=84	0.01 (-0.03, 0.04) p=0.74		
**Infection, Hospitalized**	-0.08 (-0.17, -0.00) p=0.049, n=32	0.00 (-0.07, 0.07)† p=0.99		
**Race-Center**				
	White-Forsyth		*Black-Forsyth*	
**No Infection**	-0.02 (-0.13, 0.09) p=0.75, n=731	*Reference*	0.34 (-0.20, 0.87) p=0.22, n=65	*Reference*
**Infection, not Hospitalized**	0.00 (-0.13, 0.12) p=0.97, n=45	0.02 (-0.04, 0.07) p=0.57	0.35 (-0.24, 0.94) p=0.25, n=3	0.01 (-0.18, 0.21) 0.02 p=0.89

© 2025 Demmer RT et al. *JAMA Network Open*.

**Infection, Hospitalized**	-0.05 (-0.19, 0.09) p=0.50, n=18	-0.03 (-0.12, 0.06) † p=0.51	0.02 (-0.55, 0.60)* p=0.93, n=5	-0.31 (-0.57, -0.06) p=0.017
	White-Minneapolis		*Black-Jackson*	
**No Infection**	-0.11 (-0.20, -0.01) p=0.034, n=994	*Reference*	-0.07 (-0.16, 0.02) p=0.13, n=617	*Reference*
**Infection, not Hospitalized**	-0.08 (-0.20, 0.03) p=0.16, n=40	0.02 (-0.03, 0.08) p=0.41	-0.10 (-0.21, 0.00) p=0.056, n=34	-0.03 (-0.10, 0.03) p=0.29
**Infection, Hospitalized**	-0.09 (-0.21, 0.04) p=0.16, n=22	0.02 (-0.06, 0.10) † p=0.66	-0.25 (-0.38, -0.12)* p<0.001, n=28	-0.18 (-0.28, -0.08) p<0.001
	White-Washington			
**No Infection**	-0.10 (-0.19, -0.02) p=0.015, n=811	*Reference*		
**Infection, not Hospitalized**	-0.08 (-0.18, 0.01) p=0.069, n=82	0.02 (-0.02, 0.06) p=0.28		
**Infection, Hospitalized**	-0.15 (-0.25, -0.05)* p=0.004, n=30	-0.05 (-0.06, -0.04)† p=0.11		
**APOE ε4 Alleles**				
	0 Alleles		*≥1 Alleles*	
**No Infection**	-0.07 (-0.12, -0.03) p=0.002, n=2,383	*Reference*	-0.10 (-0.19, -0.01) p=0.027, n=835	*Reference*
**Infection, not hospitalized**	-0.07 (-0.12, -0.02) p=0.008, n=155	0.00 (-0.02, 0.03) p=0.87	-0.11 (-0.22, 0) p=0.045, n=49	-0.01 (-0.07, 0.05) p=0.73
**Infection, hospitalized**	-0.11 (-0.17, -0.05) p<0.001, n=79	-0.04 (-008, -0.00) p=0.042	-0.21 (-0.33, -0.08) p=0.002, n=24	-0.11 (-0.21, -0.01) p=0.033
**Diabetes Status**				
	No Diabetes		*Diabetes*	
**No Infection**	-0.07 (-0.12, -0.02) p=0.006, n=2,197	*Reference*	-0.09 (-0.16, -0.02) p=0.008, n=1,021	*Reference*
**Infection, not hospitalized**	-0.06 (-0.12, -0.01) p=0.027, n=137	0.01 (-0.02, 0.03) p=0.72	-0.11 (-0.19, -0.03) p=0.007, n=67	-0.02 (-0.06, 0.03) p=0.48
**Infection, hospitalized**	-0.08 (-0.15, -0.01) p=0.024, n=54	-0.01 (-0.06, 0.04) p=0.71	-0.22 (-0.31, -0.13)* p<0.0001, n=49	-0.13 (-0.20, -0.07)† p<0.0001

Results adjusted for age, sex, race-center, education, smoking, alcohol use, APOE ε4 carrier status, BMI, blood pressure, history of hypertension, coronary heart disease, diabetes, stroke. 'Annual Change' parameter estimates represent the overall annualized cognitive score change rate by infection status derived from the main effect of time + the interaction between time and infection status. 'Excess Annual Change' parameter estimates represent the excess change in cognitive scores associated with infection with or without hospitalization and are derived from the interaction of follow-up time with infection.
*p-value for annual change in cognitive score between participants with hospitalized infection versus infection without hospitalization<0.05.
†p-value <0.05 for between subgroup comparison of excess change in cognitive score estimates for hospitalized infection; the reference subgroups for comparison were Age <80, Female, <High School, Black-Forsyth, 0 APOE ε4 Alleles, No Diabetes.

© 2025 Demmer RT et al. *JAMA Network Open*.

eFigure 1. Flowchart of Participants Selected for, and Excluded From, the Analysis Among Those Who Attended Either Visit 6 or Visit 7 (2016-2019) and Contributed a Prepandemic Cognitive Assessment

A flow chart from baseline to visit 5 has been previously published[27].

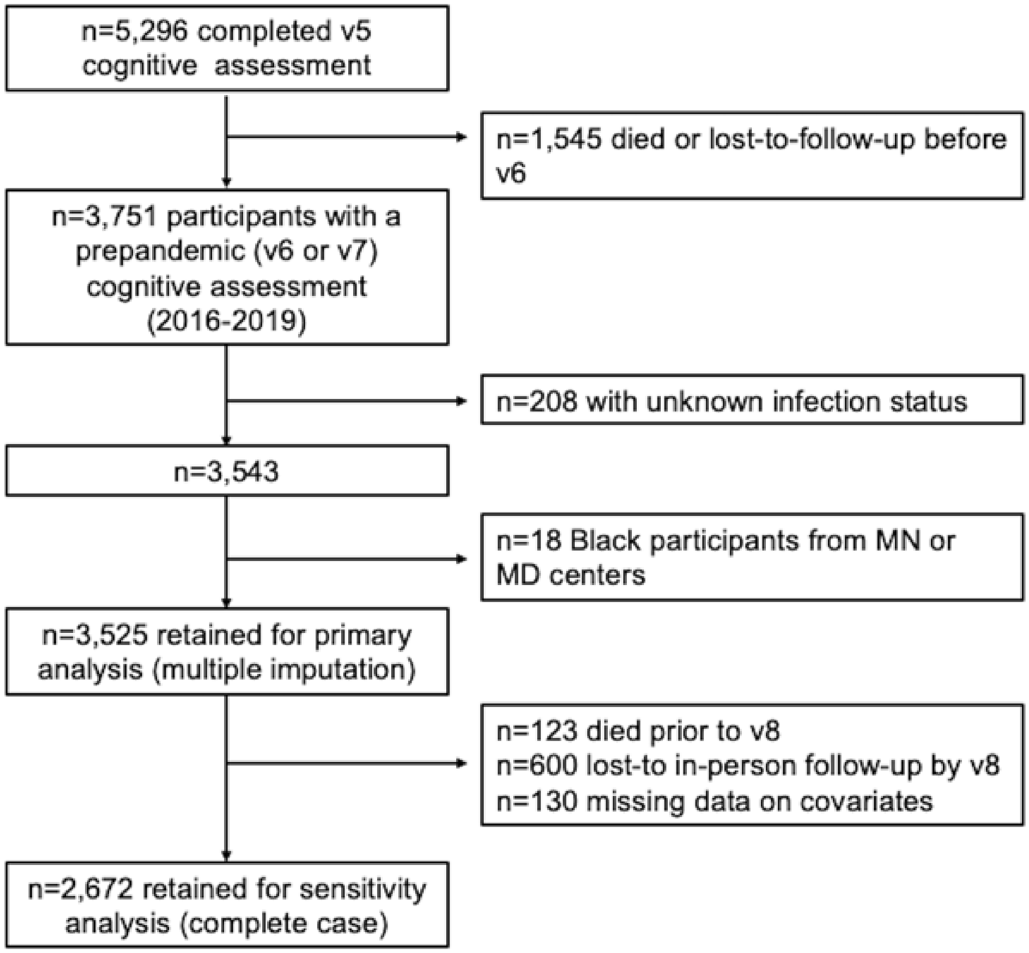

© 2025 Demmer RT et al. *JAMA Network Open*.

eFigure 2. Upset Plot Summarizing the Overlap of Sources of Information Used to Establish the Infection Exposure Definition

The upset plot visualizes the intersection of multiple sets. Rows correspond to the sets (i.e., the different methods used to identify infections). Columns represent the intersection of sets (i.e., the number of infections for which a unique combination of sets intersect). For example, the horizontal bar in the first row indicates that an ICD code contributed to the identification of n=80 infections. The first column indicates that n=62 infections were determined by a participant self-report only and the 4th column indicates that n=29 infections were identified by both an ICD code and documentation of a hospitalization via a medical record review. Overall, n=135 (44%) of 307 infections were identified by at least 2 sources. DBS=dried blood spot; MR=medical record.

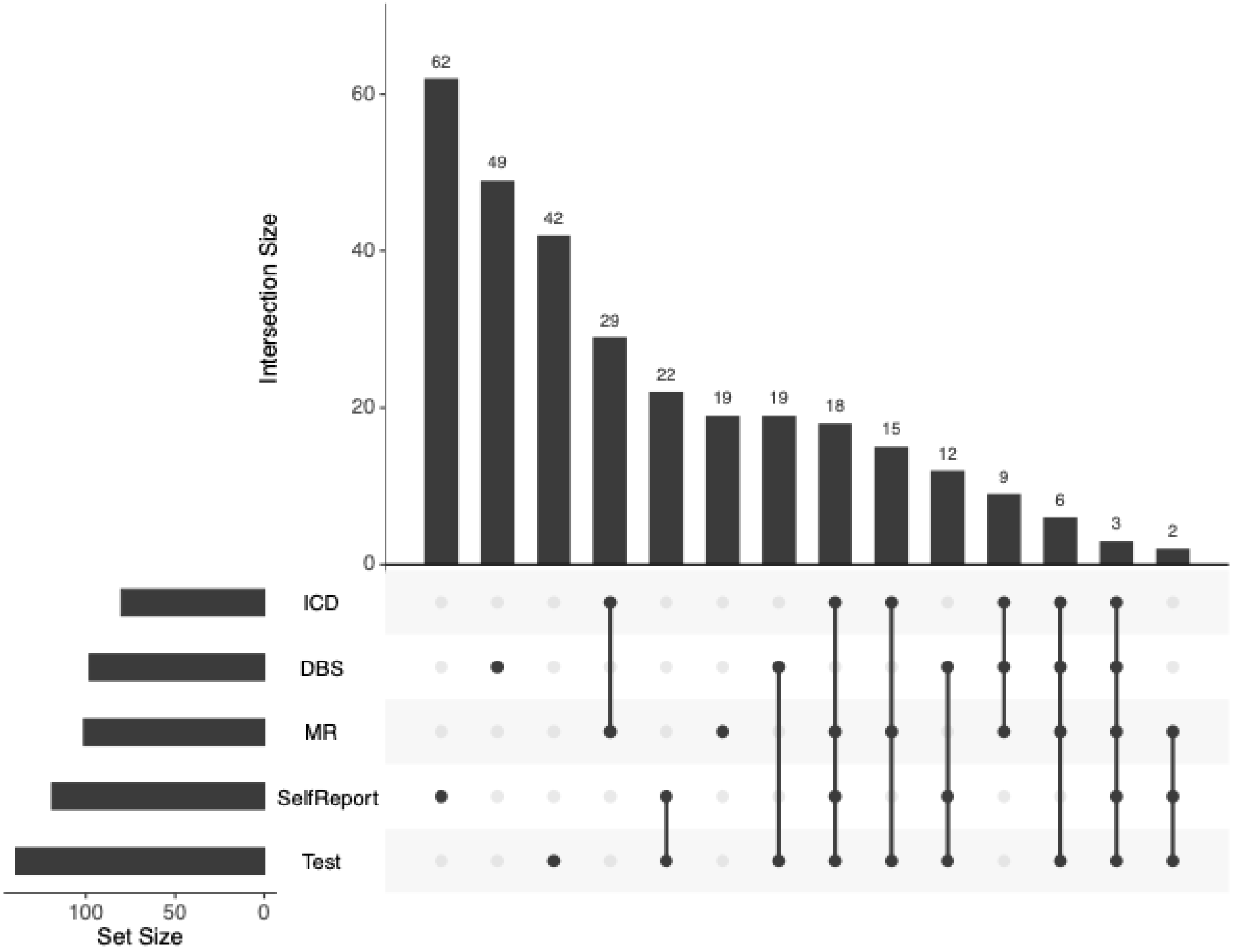

© 2025 Demmer RT et al. *JAMA Network Open*.

eReferences

1. Folstein MF, Folstein SE, McHugh PR. "Mini-mental state". A practical method for grading the cognitive state of patients for the clinician. *J Psychiatr Res*. Nov 1975;12(3):189-98. doi:10.1016/0022-3956(75)90026-6
2. Blessed G, Tomlinson BE, Roth M. The association between quantitative measures of dementia and of senile change in the cerebral grey matter of elderly subjects. *Br J Psychiatry*. Jul 1968;114(512):797-811. doi:10.1192/bjp.114.512.797
3. Blessed G, Tomlinson BE, Roth M. Blessed-Roth Dementia Scale (DS). *Psychopharmacol Bull*. 1988;24(4):705-8.
4. Wechsler D. *Wechsler adult intelligence scale-III*. The Psychological Corporation; 1997.
5. Williams BW, Mack W, Henderson VW. Boston Naming Test in Alzheimer's disease. *Neuropsychologia*. 1989;27(8):1073-9. doi:10.1016/0028-3932(89)90186-3
6. Benton A, Hamsher KD. *Multilingual Aphasia Examination*. University of Iowa; 1976.
7. Reitan R. Validity of the trail making test as an indicator of organic brain damage. *Percept Mot Skills*. 1958;8:271-276.
8. Ryan J, Lopez S. *Wechsler adult intelligence scale-III. Understanding psychological assessment. Perspectives on individual differences.* Kluwer Academic/Plenum Publishers; 2001.
9. Knopman DS, Ryberg S. A verbal memory test with high predictive accuracy for dementia of the Alzheimer type. *Arch Neurol*. Feb 1989;46(2):141-5. doi:10.1001/archneur.1989.00520380041011
10. Ricker JH, Axelrod BN. Analysis of an Oral Paradigm for the Trail Making Test. *Assessment*. Mar 1994;1(1):47-52. doi:10.1177/107319119400100107
11. Morris JC, Heyman A, Mohs RC, et al. The Consortium to Establish a Registry for Alzheimer's Disease (CERAD). Part I. Clinical and neuropsychological assessment of Alzheimer's disease. *Neurology*. Sep 1989;39(9):1159-65. doi:10.1212/wnl.39.9.1159
12. Gross AL, Power MC, Albert MS, et al. Application of latent variable methods to the study of cognitive decline when tests change over time. *Epidemiology*. 2015;26(6):878-887.
13. Rawlings AM, Bandeen-Roche K, Gross AL, et al. Factor structure of the ARIC-NCS Neuropsychological Battery: An evaluation of invariance across vascular factors and demographic characteristics. *Psychol Assess*. Dec 2016;28(12):1674-1683. doi:10.1037/pas0000293
14. Gross AL, Power MC, Albert MS, et al. Application of Latent Variable Methods to the Study of Cognitive Decline When Tests Change over Time. *Epidemiology*. Nov 2015;26(6):878-87. doi:10.1097/EDE.0000000000000379
15. Crane PK, Narasimhalu K, Gibbons LE, et al. Item response theory facilitated cocalibrating cognitive tests and reduced bias in estimated rates of decline. *J Clin Epidemiol*. Oct 2008;61(10):1018-27 e9. doi:10.1016/j.jclinepi.2007.11.011
16. Gibbons RD, Perraillon MC, Kim JB. Item Response Theory Approaches to Harmonization and Research Synthesis. *Health Serv Outcomes Res Methodol*. Dec 1 2014;14(4):213-231. doi:10.1007/s10742-014-0125-x
17. Oelsner EC, Krishnaswamy A, Balte PP, et al. Collaborative Cohort of Cohorts for COVID-19 Research (C4R) Study: Study Design. *Am J Epidemiol*. Jun 27 2022;191(7):1153-1173. doi:10.1093/aje/kwac032
18. Styer LM, Hoen R, Rock J, et al. High-Throughput Multiplex SARS-CoV-2 IgG Microsphere Immunoassay for Dried Blood Spots: A Public Health Strategy for Enhanced Serosurvey Capacity. *Microbiol Spectr*. Sep 3 2021;9(1):e0013421. doi:10.1128/Spectrum.00134-21
19. Hughes CP, Berg L, Danziger WL, Coben LA, Martin RL. A new clinical scale for the staging of dementia. *Br J Psychiatry*. Jun 1982;140:566-72. doi:10.1192/bjp.140.6.566

© 2025 Demmer RT et al. *JAMA Network Open*.

20. Pfeffer RI, Kurosaki TT, Harrah CH, Jr., Chance JM, Filos S. Measurement of functional activities in older adults in the community. *J Gerontol*. May 1982;37(3):323-9. doi:10.1093/geronj/37.3.323
21. Knopman DS, Roberts RO, Geda YE, et al. Validation of the telephone interview for cognitive status-modified in subjects with normal cognition, mild cognitive impairment, or dementia. *Neuroepidemiology*. 2010;34(1):34-42. doi:10.1159/000255464
22. Welsh KA, Breitner JCS, Magruderhabib KM. Detection of Dementia in the Elderly Using Telephone Screening of Cognitive Status. *Neuropsy Neuropsy Be*. Apr 1993;6(2):103-110.
23. Galvin JE, Roe CM, Powlishta KK, et al. The AD8: a brief informant interview to detect dementia *Neurology*. 2005;65(4):559-564.
24. Callahan CM, Unverzagt FW, Hui SL, Perkins AJ, Hendrie HC. Six-item screener to identify cognitive impairment among potential subjects for clincial research. *Medical Care*. 2002;40(9):771-781.
25. Alonso A, Mosley TH, Jr., Gottesman RF, Catellier D, Sharrett AR, Coresh J. Risk of dementia hospitalisation associated with cardiovascular risk factors in midlife and older age: the Atherosclerosis Risk in Communities (ARIC) study. *J Neurol Neurosurg Psychiatry*. Nov 2009;80(11):1194-201. doi:10.1136/jnnp.2009.176818
26. Schneider AL, Gottesman RF, Mosley T, et al. Cognition and incident dementia hospitalization: results from the atherosclerosis risk in communities study. *Neuroepidemiology*. 2013;40(2):117-24. doi:10.1159/000342308
27. Knopman DS, Pike JR, Gottesman RF, et al. Patterns of cognitive domain abnormalities enhance discrimination of dementia risk prediction: The ARIC study. *Alzheimers Dement*. Jul 2024;20(7):4559-4571. doi:10.1002/alz.13876

© 2025 Demmer RT et al. *JAMA Network Open*.
