# Supplementary material for: COVID-19 and Cognitive Change in a Community-Based Cohort
Source: JAMA Netw Open. 2025 Jun 30;8(6):e2518648. doi: 10.1001/jamanetworkopen.2025.18648 (PMC12210084; doi:10.1001/jamanetworkopen.2025.18648)
Supplement: Supplement 2. — Data Sharing Statement [file jamanetwopen-e2518648-s002.pdf]

Data Sharing Statement

Demmer. COVID-19 and Accelerated Cognitive Change in a Community-Based Cohort. *JAMA Netw Open*. Published July 02, 2025. doi:10.1001/jamanetworkopen.2025.18648

Data
Data available: Yes
Data types: Deidentified participant data, Data dictionary
How to access data: data and a data dictionary are available upon request by qualified individuals with human subjects research training and after approval of a data use proposal.
When available: beginning date: 01-01-2026

Supporting Documents
Document types: None

Additional Information
Who can access the data: Qualified individuals with human subjects research training and the necessary expertise to frame and test scientific hypotheses.
Types of analyses: Data are available for specified purposes required to be outlined in a data use protocol.
Mechanisms of data availability: A data use agreement.
